# Links Between Communication and Relationship Satisfaction Among Patients With Cancer and Their Spouses: Results of a Fourteen-Day Smartphone-Based Ecological Momentary Assessment Study

**DOI:** 10.3389/fpsyg.2018.01843

**Published:** 2018-10-10

**Authors:** Shelby L. Langer, Joan M. Romano, Michael Todd, Timothy J. Strauman, Francis J. Keefe, Karen L. Syrjala, Jonathan B. Bricker, Neeta Ghosh, John W. Burns, Niall Bolger, Blair K. Puleo, Julie R. Gralow, Veena Shankaran, Kelly Westbrook, S. Yousuf Zafar, Laura S. Porter

**Affiliations:** ^1^College of Nursing and Health Innovation, Arizona State University, Phoenix, AZ, United States; ^2^Department of Psychiatry and Behavioral Sciences, University of Washington School of Medicine, Seattle, WA, United States; ^3^Department of Psychology, Duke University, Durham, NC, United States; ^4^Department of Psychiatry and Behavioral Sciences, Duke University School of Medicine, Durham, NC, United States; ^5^Clinical Research Division, Fred Hutchinson Cancer Research Center, Seattle, WA, United States; ^6^Public Health Sciences Division, Fred Hutchinson Cancer Research Center, Seattle, WA, United States; ^7^Department of Behavioral Sciences, Rush Medical College, Rush University, Chicago, IL, United States; ^8^Department of Psychology, Columbia University, New York, NY, United States; ^9^Medical Oncology, University of Washington School of Medicine, Seattle, WA, United States; ^10^Department of Medicine, Duke University School of Medicine, Durham, NC, United States

**Keywords:** dyadic coping, cancer, spouse, partner, holding back, couples, emotional expression

## Abstract

Cancer treatment poses significant challenges not just for those diagnosed with the disease but also for their intimate partners. Evidence suggests that couples' communication plays a major role in the adjustment of both individuals and in the quality of their relationship. Most descriptive studies linking communication to adjustment have relied on traditional questionnaire methodologies and cross-sectional designs, limiting external validity and discernment of temporal patterns. Using the systemic-transactional model of dyadic coping as a framework, we examined intra- and inter-personal associations between communication (both enacted and perceived) and relationship satisfaction (RS) among patients with stage II–IV breast or colorectal cancer and their spouses (*N* = 107 couples). Participants (mean age = 51, 64.5% female patients, and 37.4% female spouses) independently completed twice-daily ecological momentary assessments (EMA) via smartphone for 14 consecutive days. Items assessed RS and communication (expression of feelings, holding back from expression, support and criticism of partner, and parallel ratings of partner behavior). Linear mixed models employing an Actor Partner Interdependence Model were used to examine concurrent, time-lagged, and cross-lagged associations between communication and RS. Expressing one's feelings was unassociated with RS. Holding back from doing so, in contrast, was associated with lower RS for both patients and spouses in concurrent models. These effects were both intrapersonal and interpersonal, meaning that when individuals held back from expressing their feelings, they reported lower RS and so too did their partner. Giving and receiving support were associated with one's own higher RS for both patients and spouses in concurrent models, and for patients in lagged models. Conversely, criticizing one's partner and feeling criticized were maladaptive, associated with lower RS (own and in some cases, partner's). Cross-lagged analyses (evening RS to next-day afternoon communication) yielded virtually no effects, suggesting that communication may have a stronger influence on short-term RS than the reverse. Findings underscore the importance of responsive communication, more so than expression *per se*, in explaining both concurrent and later relationship adjustment. In addition, a focus on holding back from expressing feelings may enhance the understanding of RS for couples coping with cancer.

## Introduction

A diagnosis of cancer poses great challenges for both patients and their loved ones. Patients with cancer often experience significant emotional distress including anxiety and worry, depression, and fears of disease progression and death (Syrjala and Yi, 2018). Many patients also report multiple disease and treatment-related side effects including fatigue, pain, cognitive impairment, and sexual dysfunction (Bower, 2008; Syrjala and Yi, 2018). These problems can limit patients' ability to perform many of their usual family and workplace responsibilities, thus disrupting their role functioning in important areas (Zebrack, 2000; Syrjala et al., 2004). For the many patients who are married or in committed partnerships, cancer also poses formidable challenges for their significant others and relationships (Carlson et al., 2000). Partners of individuals with cancer experience an array of psychological difficulties (Baider et al., 1996; Bishop et al., 2007). Research suggests that patients' and partners' levels of psychological distress are moderately correlated (Hagedoorn et al., 2008), and that some partners suffer more distress than do patients (Given and Given, 1992; Langer et al., 2003). In addition, patients with advanced cancer as well as their partners report more distress, role restrictions and physical difficulties than do those coping with early stage cancers (Weitzner et al., 1999; Badr et al., 2013). These impacts to both patients and partners, and the need for mutual responsiveness and support, have led to the description of cancer as a “we-disease” (Acitelli and Badr, 2005; Kayser et al., 2007).

One way in which couples can support each other is through effective communication (Kayser et al., 2007). Accumulating evidence indicates that couples' ability to communicate effectively plays a major role in the psychological adjustment of patients and partners and the quality of their relationship (Baucom et al., 2012). Specifically, communication behaviors that are associated with better patient and partner adjustment include open discussion of cancer-related concerns (often referred to as disclosure), and the ability to listen and respond supportively to one's partner. Maladaptive communication behaviors include holding back from disclosure and avoiding or responding negatively to one's partner's disclosure. A variety of questionnaires have been developed to assess adaptive and maladaptive communication behavior, including those assessing *disclosure* (Laurenceau et al., 2005) and *holding back* from disclosure (Porter et al., 2005); *protective buffering*, which is defined as hiding concerns or dispiriting information from one's partner, denying one's worries, or capitulating in order to avoid conflict (Hagedoorn et al., 2000); and *social constraints*, which are perceptions that the partner's responses to one's own disclosures are avoidant, discouraging or disapproving (Lepore and Revenson, 2007). In general, individuals who report low levels of disclosure, or high levels of holding back, protective buffering or social constraints also report greater psychological distress and poorer relationship functioning (Suls et al., 1997; Kayser et al., 1999; Hagedoorn et al., 2000; Kuijer et al., 2000; Porter et al., 2005; Manne et al., 2007, 2014b, 2015; Hinnen et al., 2009; Langer et al., 2009; Traa et al., 2015).

Even in the context of satisfying relationships, couples may experience difficulty communicating about cancer-related issues (Lichtman and Wood, 1987; Pistrang and Barker, 1995), for a number of reasons. First, both patients and partners may feel overwhelmed with the logistical, physical, and emotional challenges associated with cancer, including attending medical appointments, making medical decisions, providing emotional and physical assistance, and coping with treatment side effects and their own emotional distress. They may thus find it difficult to make time for meaningful conversation or to articulate specific concerns. Second, both patients and partners often mistakenly believe that it will be harmful or upsetting for patients to discuss their cancer, worries, or any negative aspects of the situation, and that the partners' role is to be continually cheerful and optimistic (Peters-Golden, 1982). Third, patients and partners often avoid discussing sensitive issues such as sexual functioning or disease progression and death (Porter et al., 2005; Reese et al., 2010).

Communication between patients with cancer and their intimate partners can be conceptualized in terms of the systemic-transactional model of stress and coping in couples (Bodenmann, 1995). This model builds upon earlier conceptions of individual-level stress and coping (Lazarus and Folkman, 1984) which focused on an individual's appraisal of the threat of the stressor and the extent to which s/he feels capable of coping with it. Bodenmann (1995, 2005) extended this model to dyads, noting that stress affects not just individual members of the dyad, but both parties, either directly or indirectly. If the stress is direct, both dyad members are affected concurrently, for example, if the couple's roof is leaking and needs immediate repair. If the stress is indirect, the stress experienced by one dyad member subsequently ends up impacting the other because the first is unable to adequately handle the stressor. For example, intense workplace demands might cause one dyad member to have to routinely stay late at the office, hence impeding his/her ability to pick up the couple's child after school, as was heretofore his/her usual practice. The other dyad member is thus affected. Whether or not both partners are affected directly, they can engage in dyadic coping which is conceptualized as “a systemic coping pattern, enrolling both partners in a symmetrical, complementary or occasionally asymmetric way” (Bodenmann, 1995).

Bodenmann (2005) noted that prior to the introduction of the systemic transactional model, research on stress and couples took one of three forms: (1) individual-level coping efforts in the context of a partnered relationship; (2) interactions between each dyad member's individual-level coping efforts; and (3) coping efforts on the part of one dyad member to promote better functioning for the other and the relationship as a whole. Protective buffering is mentioned as an example of the latter. One dyad member might act more positive than s/he feels so as to not upset the other. These approaches were distinguished from dyadic coping, in which *both* partners work conjointly to manage a stressor. Dyadic coping may be particularly relevant in the context of cancer because it is a common stressor that requires coordinated coping and management efforts, as well as mutual responsiveness (Kayser et al., 2007).

The present study focuses on two specific forms of dyadic coping: emotion-focused positive supportive dyadic coping and negative dyadic coping (Bodenmann, 2005), both particularly relevant to dyadic communication in couples dealing with cancer. According to the systemic transactional model, stress experienced by one dyad member may or may not be communicated to the other. Stress that *is* communicated in some way (verbally or non-verbally) is encoded and interpreted by the other dyad member, who may or may not respond. If the response conveys empathic understanding, validation and support, emotion-focused positive supportive dyadic coping is said to be occurring. If the response conveys hostility or ambivalence, negative dyadic coping is said to be occurring. Research on these specific elements of dyadic coping indicate positive associations between emotion-focused positive supportive dyadic coping and relationship satisfaction (RS), and inverse associations between negative dyadic coping and RS (Traa et al., 2015).

In addition to the elements of the systemic transactional model described above, the present study also incorporated the concept of holding back as an important element of potentially dysfunctional communication patterns in couples dealing with cancer. Holding back is seen as not simply the lack of expression or disclosure, but as an intentional or active attempt to suppress communication about difficult topics, a strategic behavior pattern that involves and affects both partners (Porter et al., 2005). Prior research has demonstrated associations between holding back and patient and partner maladjustment in the context of cancer (Porter et al., 2005; Edmond et al., 2013; Manne et al., 2014a). In addition, while the systemic transactional model focuses on stress communication specifically, we adopted a broader approach, including disclosures both related and unrelated to cancer, given the difficulty of defining a priori which communications might be stressful in the context of cancer.

In the present study, we sought to examine associations between communication (or holding back from communication), emotion-focused positive supportive dyadic coping, and negative dyadic coping at a given point in time and RS at (a) the same time and (b) a later point in time using ecological momentary assessment (EMA) methods. To date, most descriptive studies linking communication to adjustment in cancer have relied on cross-sectional designs which limit our ability to discern temporal patterns and do not allow for optimal tests of hypotheses regarding transactional causality in dyadic interactions. Few longitudinal studies exist, particularly those in which data are collected in real-time and in naturalistic settings. EMA offers a number of advantages. First, it minimizes recall biases inherent in global, retrospective measures which require participants not just to remember but also to summarize their behavior (e.g., “To what extent did you talk to your partner about cancer-related concerns during the past week?”). When considering such questions, individuals use a variety of heuristics to estimate their answers, leading to systematic biases influenced, for example, by current mood (Shiffman et al., 2008). Second, compared to both global self-report and laboratory-based assessments, EMA increases ecological validity (as participants are reporting in their real-world environment) and enables examination of within-day as well as day-to-day variations in behavior and experiences. Finally, the longitudinal nature of EMA data can be used to examine temporal sequences of behaviors and experiences (Shiffman et al., 2008). Thus, like laboratory-based observational data, EMA enables the study of both within-person and within-couple effects and directionality of such. However, it does so in the participants' natural environment and over a period of days or weeks.

At least two prior studies used a daily diary approach to explore communication among persons with breast cancer and their partners. One study (Pasipanodya et al., 2012) found that patients who reported higher perceived social constraints at baseline shared fewer positive and negative events with their partner on a daily basis, and that patients *and* partners who reported higher social constraints at baseline reported higher levels of negative mood and decreased relationship functioning on a daily basis. In the second study (Badr et al., 2013), women with metastatic breast cancer and their partners reported each day on the degree to which they avoided talking to each other about the *patient's* cancer-related concerns. Results indicated that partner but not patient avoidance varied from day to day, and that greater partner avoidance was related to increased patient negative affect the following day. Both of these studies had some important limitations including a lack of assessment of partner communication about their concerns, and samples of exclusively female patients, thereby confounding gender and role. The current study builds on these findings by assessing a wider range of patient and spouse communication behaviors among a sample that includes both male and female patients.

The aims of the present study were to examine intra- and inter-personal concurrent and lagged associations between communication, including patient and spouse reports of their own communication and perceptions of their partner's communication, and RS. Our specific hypotheses were derived primarily from the systemic transactional model but also reflected the addition of holding back as a potentially influential behavior. Actor and partner effects for both patients and spouses were hypothesized as follows: Expressing one's feelings and providing and receiving emotion-focused positive support would be positively associated with concurrent and later RS (both own and partner's). Conversely, being critical and feeling criticized would be inversely associated with concurrent and later RS (both own and partner's), as would holding back from expressing one's feelings. We also examined the cross-lagged effects of RS on next-day communication to explore the possibility of these reciprocal effects.

## Materials and methods

### Participants

All procedures, including screening and approach processes, were approved by the Institutional Review Boards of Arizona State University (Social Behavioral Committee) and Duke University School of Medicine (via expedited review; no subcommittee specified). Participants were recruited from two different cancer centers, the Duke Cancer Institute in Durham, NC and the Seattle Cancer Care Alliance in Seattle, WA (an alliance of the Fred Hutchinson Cancer Research Center and the University of Washington). The Institutional Review Board of Arizona State University served as the IRB of record for the Seattle site, with IRB authorization agreements held by the Fred Hutchinson Cancer Research Center and the University of Washington. All subjects gave written informed consent in accordance with the Declaration of Helsinki.

Inclusion criteria for patients were: age 18 or older; stage II–IV breast, colon, or rectal cancer; currently receiving or having received chemotherapy and/or hormone therapy; within 2 years of diagnosis of current cancer stage; life expectancy of at least 6 months per primary oncologist; married or in a committed, cohabiting relationship with someone of the same or opposite sex; and ability to speak and comprehend English. Inclusion criteria for spouses were: age 18 or older; married to or in a committed, cohabiting relationship with the patient; and ability to speak and comprehend English. Please note that the term “spouse” is used throughout but encompasses both spouses and non-married cohabiting partners. This is to avoid confusion with the term “partner” as used in the EMA item wording and in the statistical sense (partner effect).

Among patients screened for the protocol at the time of data extraction (*N* = 944), 396 were deemed initially eligible based on medical records. Among the 396, 268 were contacted, further screened, and deemed fully eligible. Among those, 136 agreed to a face-to-face study visit with their partner, a 50.7% agreement rate. The most common reasons for refusal were lack of interest in the study or simply being too busy. Twenty of the 136 scheduled appointments were beyond the time frame of analysis for the present manuscript. Among the 116 couples seen face-to-face at the time of data extraction, 115 signed consent forms (separate forms for patients and spouses). Three of the 115 couples signed consent forms for the study in general but elected not to participate in the EMA portion of the study, due to discomfort with technology (EMA questions were posed via smartphone). Five of the 112 were excluded from the current analyses because of either insufficient data provision due to technological problems (*n* = 2 couples) or because they had not yet reached the end of the EMA phase at the time of data extraction (*n* = 3 couples), yielding an analytic sample of 107 couples.

### Procedures

Patients who met initial medical inclusion criteria per medical records were sent a study brochure and letter from their primary oncologist introducing the study. This approach letter informed patients that a research team member would contact them by telephone to provide study details and gave options for patients to initiate contact themselves or to opt out. During the initial telephone contact, site research coordinators conducted further eligibility screening, including confirmation that the patient was married or in a committed and cohabiting relationship and that both patient and spouse had sufficient English comprehension and speaking ability to complete study activities.

Patients who expressed willingness to participate in the study (as a couple with their partner), were asked to identify a convenient time for a 90-min in-person visit, often scheduled around their next medical appointment to minimize additional travel to the clinic. Couples met with the site research coordinator in a private room at or near the site clinic. Written consent was obtained separately from patients and spouses following a detailed description of study procedures, risks and benefits, and assuring that any and all questions or concerns were addressed. After consent, participants completed other study activities not included in this report (e.g., a battery of psychosocial questionnaires and a cancer-related couple conversation) and then downloaded the free smartphone application.

The smartphone application was created using lifedatacorp.com, a web-based application development platform. The application is compatible with iOS and Android cell telephones. Participants used their personal phones unless they owned a different kind of phone or did not own a phone, in which case they were lent an iPod Touch device for study use. This was the case for 15 individuals. Participants who borrowed an iPod Touch completed the activity using the device as they would a smartphone. The application download was conducted in the same manner as with smartphones and application interfaces and participant views were comparable.

The lifedatacorp.com system allows users to receive questions via notification once the application has been downloaded on participants' mobile devices and they have signed up as individual users. The site research coordinators guided participants through the application download process. Upon download and required user registration, participants began receiving notifications to complete assessments twice daily for 14 days: once at 12:00 p.m. (afternoon assessment) and once at 8:00 p.m. (evening assessment). The response window was set for 2 h from the time of the first notification for both assessment periods. If participants did not begin to complete the assessment within the 2-h response window, the notification expired.

Participants received a Frequently Asked Questions user handout developed for the smartphone portion of the study and were provided with site research coordinator contact information in case of any technical difficulties. Participants were contacted 2–3 days after the application download to check in and provide assistance as needed. At the completion of the 14-day EMA, participants were sent a $75 check or gift card if they had completed 85% or more of the notifications received. If <85% of assessments were completed, participants were paid $3 per notification completed.

Demographic characteristics (age, gender, race, ethnicity, education, and income) were assessed via questionnaire using Research Electronic Data Capture (REDCap), a secure web-based tracking and on-line data acquisition system (Harris et al., 2009). REDCap was also used to administer the Dyadic Adjustment Scale, a reliable and valid measure of relationship adjustment (Spanier, 1976). Medical records were extracted to screen for eligibility, using a HIPAA waiver.

### Measures

Ecological momentary assessment items are described below.

#### Communication with partner

Participants were asked whether or not they had talked to their partner since awakening (afternoon assessment) or since the last notification (evening assessment). What constituted “talking” was not defined and therefore left up to interpretation by participants. Accordingly, participants may have counted telephone calls or text messages with their partner as conversations, but this was not measured *per se*. Those responding “no” were asked why not: I didn't have any contact with my partner; I had nothing to talk about; I didn't feel well; I didn't want to bring up topics that could be upsetting; and other. Those responding “yes” were asked a series of follow-up questions about the conversation. The first item assessed relatedness to the cancer: To what extent was this conversation related to the cancer (1 = not at all; 3 = somewhat; 5 = a lot)? The second item assessed perceived importance: How important was this conversation to you (1 = not at all; 3 = somewhat; 5 = extremely)?

Remaining items (listed in Table 1 to illustrate mapping on to the STM) assessed the extent to which, during the conversation, participants: (1) expressed their feelings, (2) held back from expressing their feelings, (3) supported their partner, and (4) criticized their partner. The latter two items were created for the study based on face validity. The former two items were adapted from the Emotional Disclosure Scale (Pistrang and Barker, 1995). This scale asks respondents to rate the extent to which they talked to their partner about each of several cancer-related concerns (disclosure) and the extent to which they held back from doing so (holding back). For the present EMA administration, ratings were made with respect to the conversation in question and not tied to specific concerns. We also administered the full Emotional Disclosure Scale at baseline (pre-EMA), as part of a larger battery of questionnaires. Correlations between the two administrations were positive in sign: *r* = 0.54, *p* < 0.001 for patients and *r* = 0.58, *p* < 0.001 for spouses [holding back]; and *r* = 0.16, *p* = 0.102 for patients and *r* = 0.08, *p* = 0.419 for spouses [disclosure]. These associations provide some support for validity, more so for the holding back item. Note that overlap is not expected to be great given that one set of ratings is necessarily cancer-related and the other is not. Parallel EMA items assessed perceptions of partner communicative behavior (Table 1), with one exception. We did not ask participants to rate the extent to which their partner held back, assuming this would have been difficult to judge. In contrast, behaviors such as criticism are presumably more readily observable.

**Table 1:** Ecological momentary items designed to assess communication and analogous constructs per the systemic transactional model (STM).

**Item**	**Parallel dyadic coping construct per the systemic transactional model**
**To what extent did you…**	
Express your feelings during this conversation*	Stress communication
Hold back from expressing your feelings	Not addressed by STM
Support your partner	Emotion-focused positive supportive dyadic coping
Criticize your partner	Emotion-focused negative dyadic coping
**To what extent did you feel that your partner…**	
Expressed his/her feelings*	Stress communication
Supported you	Emotion-focused positive supportive dyadic coping
Criticized you	Emotion-focused negative dyadic coping

*Items with an asterisk assess disclosure or emotional expression, but not stress communication per se*.

#### Relationship satisfaction (evening assessment only)

Following Auger (Auger et al., 2016), RS was assessed with a single item (item #31) from the satisfaction subscale of the Dyadic Adjustment Scale (Spanier, 1976). Specifically, participants were asked, “All things considered, what was your degree of happiness with your relationship today: extremely unhappy (1), fairly unhappy (2), a little unhappy (3), happy (4), very happy (5), extremely happy (6), or perfectly happy (7)?” Per Goodwin (Goodwin, 1992), this item is highly correlated with total adjustment scores based on the full Dyadic Adjustment Scale excluding item #31 (*r* = 0.73 and 0.67 from two different studies) and valid, i.e., able to discriminate persons classified as adjusted vs. distressed using the total scale score excluding item #31.

In the present study, we administered the full DAS to patients and spouses as part of a baseline battery of questionnaires. Baseline ratings of item #31 (general relationship happiness) were positively associated with EMA ratings of the same item (relationship happiness today), *r* = 0.49, *p* < 0.001 for patients and *r* = 0.57, *p* < 0.001 for spouses. The magnitude of these correlations suggests some degree of overlap but not complete overlap between the two measures. To characterize the degree of within-person non-independence (clustering) in patients' and spouses' RS responses, we computed intraclass correlation coefficients (ICCs), adjusting for temporal autocorrelation. ICC values for patients and spouses were nearly identical (0.60 and 0.61, respectively). These values indicate that approximately 40% of the total variation in RS was at the within-person level (i.e., across days, within individuals).

### Analysis

Descriptive statistics were used to characterize the sample with respect to demographic, clinical and relationship adjustment characteristics. Ecological momentary assessment response and completion rates, frequency of having talked to one's partner, whether or not a conversation was about the cancer, and perceived importance of a conversation were summarized separately by afternoon and evening. Correlational analyses examining associations among key study variables also were conducted (Supplemental Tables 1, 2).

**Figure 1:**
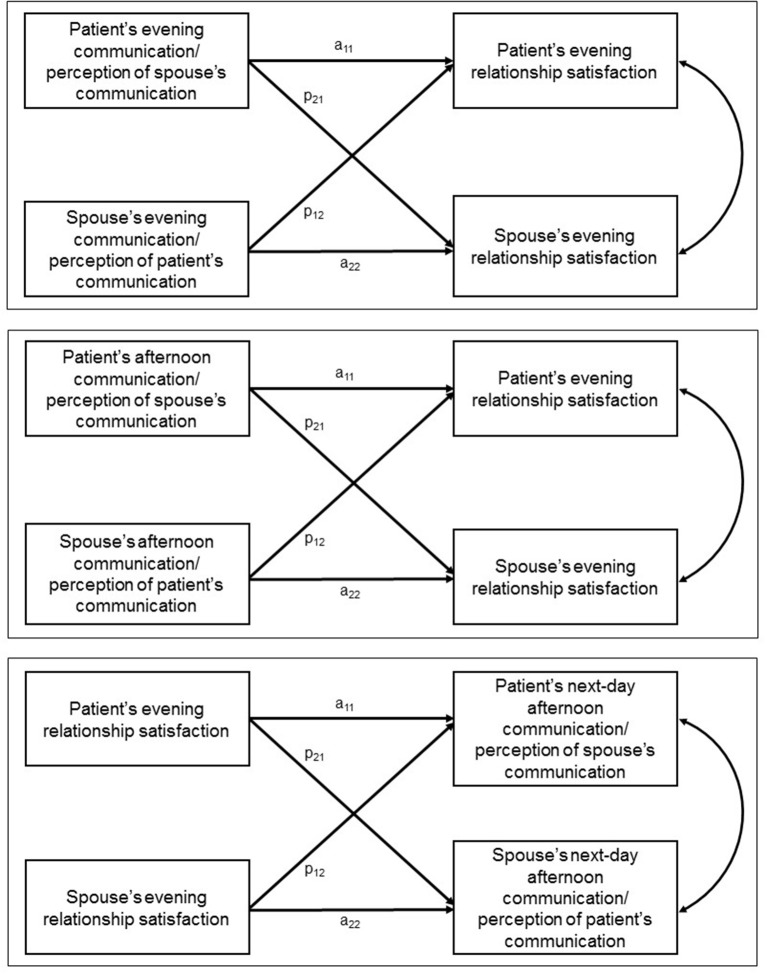
Concurrent (top panel), lagged (middle panel), and cross-lagged (bottom panel) actor-partner interdependence models.

Associations between communication variables and RS were estimated using linear mixed models within an Actor-Partner Interdependence Model (APIM) framework for dyads with distinguishable members. Three sets of APIM analyses were conducted: (1) models predicting each dyad member's RS from that member's report of his/her own communication and perceptions of his/her partner's communication on *that same evening* (concurrent models), (2) models predicting each dyad member's RS from that member's report of his/her own communication and perceptions of his/her partner's communication in the *afternoon of the same day* (lagged models), and (3) models predicting each dyad member's afternoon reports of communication and perceptions of his/her partner's communication from that member's report and his/ her partner's report of RS from *the preceding evening* (cross-lagged models). In concurrent and lagged models, four effects of substantive interest were estimated, depicted in the top two panels of Figure 1: (1) patient's reports of communication/perceived communication predicting his/her own RS (patient actor effects; coefficient a_11_); (2) spouse's reports of communication/perceived communication predicting his/her own RS (spouse actor effects; coefficient a_22_); (3) patient's reports of communication/ perceived communication predicting the spouse's RS (patient partner effects; coefficient p_21_); and (4) spouse's reports of communication/perceived communication predicting the patient's RS (spouse partner effects; coefficient p_12_). Parallel actor and partner effects were estimated in cross-lagged models, but with communication/ perceived communication being predicted from the preceding day's RS; see bottom panel of Figure 1.

In concurrent and lagged models, each dyad member's RS on a given evening was predicted from two dummy vectors coding for dyad member (with no overall intercept term) and four two-way interactions between these dummy vectors and person mean-centered evening (or afternoon) communication/perceived communication variables, each capturing one of the four effects of substantive interest. Four parallel between-person interaction terms were included. Cross-lagged models paralleled concurrent and lagged models in their general form, but with each day's afternoon communication/perceptions of communication being predicted from the preceding evening's RS. Except where noted in tables of model coefficients, intercepts and actor and partner effects were estimated as random. An AR(1) variance/covariance structure was specified for day-level residuals. Results from analyses conducted adjusting for cancer diagnosis stage (stage II vs. stage III or IV) did not differ from those based on analyses described above. Results from the more parsimonious models (i.e., those without the stage indicator) are reported here. Descriptive analyses were conducted in SPSS 24.0 and SAS/STAT 14.1. Linear mixed models were estimated via restricted maximum likelihood using all available data in SAS/STAT 14.1 PROC MIXED. Given the large number of APIM model effects analyzed, α was set at 0.01 for these tests.

## Results

### Sample characteristics

Table 2 displays demographic characteristics of patients and spouses, and clinical characteristics of patients. Patients and spouses were, on average, 50 years old. The patient sample was comprised of 64.5% females and 35.5% males. Given that most couples were heterosexual (96%), the reverse was true for spouses (37.4% female). Participants were predominantly White (87%) and non-Hispanic (96%). Sixty-three percent had earned a 4-year college degree or higher, and 57.5% reported a total household income of $100,000 or higher. Most couples were married (91.6%) and had been in their relationship for over 10 years (72%).

**Table 2:** Demographic, clinical, and relationship adjustment characteristics of the sample.

	**Patients**	**Spouses**
*N*	107	107
Age, M (SD); range	50.64 (12.33); 27–81	50.93 (13.07); 26–80
Gender, *n* (%)		
Female	69 (64.5)	40 (37.4)
Male	38 (35.5)	67 (62.6)
Race, *n* (%)		
American Indian/Alaska Native	2 (1.9)	1 (0.9)
Asian	3 (2.8)	2 (1.9)
Black of African American	6 (5.6)	6 (5.6)
White	92 (86.0)	94 (87.9)
More than one race	4 (3.7)	4 (3.7)
Ethnicity, *n* (%)
Hispanic or Latino	6 (5.6)	1 (0.9)
Not Hispanic or Latino	101 (94.4)	105 (98.1)
Unknown	0 (0.0)	1 (0.9)
Educational status, *n* (%)		
Less than high school	0 (0.0)	1 (0.9)
High school degree or GED	13 (12.1)	9 (8.4)
Some college or technical	30 (28.0)	25 (23.4)
school		
4-year college degree	33 (30.8)	41 (38.3)
Post-baccalaureate degree	31 (29.0)	30 (28.0)
Total household income, *n* (%)		
Less than $20,000	5 (4.7)	
$20,000–$39,999	3 (2.8)	
$40,000–$59,999	12 (11.2)	
$60,000–$79,999	8 (7.5)	
$80,000–$99,999	16 (15.0)	
$100,000–$120,999	15 (14.0)	
$121,000+	47 (43.9)	
Unknown	1 (0.9)	
Marital status, *n* (%)		
Married	98 (91.6)	98 (91.6)
Partnered and cohabiting	9 (8.4)	9 (8.4)
Length of relationship, *n* (%)		
1–2 years	3 (2.8)	
3–5 years	8 (7.5)	
6–10 years	19 (17.8)	
11–15 years	11 (10.3)	
11+ years	66 (61.7)	
Dyadic Adjustment Scale score,	115.51	115.00
M (SD)	(14.43)	(13.63)
Type and stage of cancer, *n* (%)		
Breast	47/107 (43.9)	–
Stage II	26/47 (55.3)	–
Stage III	7/47 (14.9)	–
Stage IV	14/47 (29.8)	–
Colon	34/107 (31.8)	–
Stage II	7/34 (20.6)	–
Stage III	8/34 (23.5)	–
Stage IV	19/34 (55.9)	–
Rectal	26/107 (24.3)	–
Stage II	6/26 (23.1)	–
Stage III	11/26 (42.3)	–
Stage IV	9/26 (34.6)	–

With regard to clinical characteristics, 44% of patients had been diagnosed with breast cancer, 32% with colon cancer, and 24% with rectal cancer. Across diagnostic groups, 64% were coping with advanced cancer, stage III or IV. The breast cancer group was characterized by more lower stage disease (55% stage II) whereas the colon cancer group was characterized by more higher stage disease (56% stage IV). The rectal group was more evenly distributed in this respect (23% stage II, 42% stage III, and 35% stage IV).

### Ecological momentary assessment feasibility and adherence

Table 3 displays descriptive characteristics of the smartphone-derived EMA data. Across 107 patients and spouses and both afternoon and evening periods, a total of 5,784 notifications were sent. Of these, 5,232 were responded to (90.5%) and 5,136 were completed (88.8%). These values were similar across patients and spouses and afternoon and evening assessment time points. Among those responding to the afternoon notification, 78.9% had conversed with their partner since awakening. Among those responding to the evening notification, 85.8% had conversed with their partner since the last assessment. The most common reason for not having conversed with one's partner was simply not having had contact with him or her (67.9% in the afternoon and 58.8% in the evening). Conversations were deemed relatively important in nature, mean ratings = 3.17 and 3.24 for afternoon and evening, respectively, on the 1–5 (not at all—extremely) scale, but were often not about the cancer. Seventy-one percent of conversations reported in the afternoon were rated as 1 or not at all about the cancer, as were 69% of conversations reported in the evening. Subsequent (concurrent and lagged) analyses were not examined separately as a function of conversation importance or cancer-relatedness due to limited power.

**Table 3:** Descriptive statistics of smartphone-gathered ecological momentary assessment data (*N* = 107 couples).

	**Patient**	**Spouse**	**Total**
Total number of notifications sent, *n*	2,893	2,891	5,784
Total number of notifications responded to/ notifications sent, *n* (%)	2,629 (90.9)	2,603 (90.0)	5,232 (90.5)
Total number of notifications completed/ notifications sent, *n* (%)	2,592 (89.6)	2,544 (88.0)	5,136 (88.8)
**Afternoon notifications only**			
Number of notifications sent	1,414	1,409	2,823
Number of notifications responded to/ notifications sent, n (%)	1,270 (89.8)	1,270 (90.1)	2,540 (90.0)
Number of notifications completed/ notifications sent, n (%)	1,251 (88.5)	1,246 (88.4)	2,497 (88.5)
Conversed with partner since awakening, n	988	1,016	2,004
Did not converse with partner since awakening, n	277	246	523
Among those who did not converse with partner since awakening, reasons why, *n*/responded to item (%)			
I didn't have any contact with my partner	194/277 (70.0)	160/244 (65.6)	354/521 (67.9)
I had nothing to talk about	18/277 (6.5)	17/244 (7.0)	35/521 (6.7)
I didn't feel well	9/277 (3.2)	1/244 (0.4)	10/521 (1.9)
I didn't want to bring up topics that could be upsetting	3/277 (1.1)	1/244 (0.4)	4/521 (0.8)
Other	53/277 (19.1)	65/244 (26.6)	118/521 (22.6)
Importance of conversation, M (SD) on 1-5 scale	3.15 (1.18)	3.18 (1.15)	3.17 (1.16)
Extent to which conversation was related to cancer, M (SD) on 1–5 scale	1.63 (1.18)	1.70 (1.24)	1.67 (1.21)
Extent to which conversation was related to cancer, n/ responded to item (%)			
Rating of 1 (not at all)	715/978 (73.1)	705/1,014 (69.5)	1,420/1,992 (71.3)
Rating of 2	66/978 (6.7)	93/1,014 (9.2)	159/1,992 (8.0)
Rating of 3 (somewhat)	96/978 (9.8)	102/1,014 (10.1)	198/1,992 (9.9)
Rating of 4	44/978 (4.5)	39/1,014 (3.8)	83/1,992 (4.2)
Rating of 5 (a lot)	57/978 (5.8)	75/1,014 (7.4)	132/1,992 (6.6)
**Evening notifications only**			
Number of notifications sent	1,479	1,482	2,961
Number of notifications responded to/ notifications sent, *n* (%)	1,359 (91.9)	1,333 (89.9)	2,692 (90.9)
Number of notifications completed/ notifications sent, *n* (%)	1,341 (90.7)	1,298 (87.6)	2,639 (89.1)
Conversed with partner since last notification, *n*	1,158	1,153	2,311
Did not converse with partner since last notification, *n*	198	166	364
Among those who did not converse with partner since last notification, reasons why, *n*/responded to item (%)			
I didn't have any contact with my partner	112/196 (57.1)	99/163 (60.7)	211/359 (58.8)
I had nothing to talk about	19/196 (9.7)	18/163 (11.0)	37/359 (10.3)
I didn't feel well	18/196 (9.2)	1/163 (0.6)	19/359 (5.3)
I didn't want to bring up topics that could be upsetting	1/196 (0.5)	1/163 (0.6)	2/359 (0.6)
Other	46/196 (23.5)	44/163 (27.0)	90/359 (25.1)
Importance of conversation, *M* (SD) on 1–5 scale	3.24 (1.16)	3.24 (1.09)	3.24 (1.13)
Extent to which conversation was related to cancer, *M* (SD) on 1–5 scale	1.70 (1.23)	1.73 (1.23)	1.72 (1.23)
Extent to which conversation was related to cancer, *n*/responded to item (%)			
Rating of 1 (not at all)	811/1,153 (70.3)	771/1,143 (67.5)	1,582/2,296 (68.9)
Rating of 2	83/1,153 (7.2)	110/1,143 (9.6)	193/2,296 (8.4)
Rating of 3 (somewhat)	123/1,153 (10.7)	136/1,143 (11.9)	259/2,296 (11.3)
Rating of 4	62/1,153 (5.4)	49/1,143 (4.3)	111/2,296 (4.8)
Rating of 5 (a lot)	74/1,153 (6.4)	77/1,143 (6.7)	151/2,296 (6.6)

### Concurrent analyses

Table 4 displays coefficients, standard errors, and *p*-values from linear mixed models predicting evening RS from same-day evening communication. Intercepts represent the adjusted sample means for RS for patients and spouses (second and third column, respectively). Labels for patient- and spouse-specific actor and partner effect coefficients (e.g., a_11_) correspond to those in the top panel of Figure 1. In what follows, we highlight statistically significant (*p* < 0.01) effects.

**Table 4:** Mixed model regression coefficients (standard errors) and *p*-values from concurrent analyses: evening communication predicting evening relationship satisfaction.

	**Intercept**		**Actor effect**		**Partner effect**	
**Predictor**	**Patient**	**Spouse**	**Patient (a_11_)**	**Spouse (a_22_)**	**Patient (p_21_)**	**Spouse (p_12_)**
**To what extent did you…**						
Express your feelings^a^	5.201 (0.099)	5.126 (0.096)	0.057 (0.033) *p* = 0.085	0.045 (0.030) *p* = 0.136	−0.014 (0.026) *p* = 0.593	0.030 (0.027) *p* = 0.263
Hold back from expressing your feelings	5.216 (0.094)	5.136 (0.090)	−**0.144** (0.041) *p* < 0.001	−**0.206** (0.054) *p* < 0.001	−**0.117** (0.039) *p* = 0.004	−**0.142** (0.044) *p* = 0.002
Support your partner^a^	5.223 (0.087)	5.126 (0.085)	**0.219** (0.037) *p* < 0.001	**0.334** (0.052) *p* < 0.001	0.065 (0.034) *p* = 0.053	**0.117** (0.032) *p* < 0.001
Criticize your partner	5.262 (0.091)	5.171 (0.087)	−**0.272** (0.065) *p* < 0.001	−**0.225** (0.053) *p* < 0.001	−0.066 (0.060) *p* = 0.276	−**0.187** (0.066) *p* = 0.009
**To what extent did you feel that your partner…**						
Expressed his/ her feelings^a^	5.203 (0.097)	5.139 (0.092)	**0.127** (0.032) *p* < 0.001	0.076 (0.035) *p* = 0.035	0.010 (0.029) *p* = 0.734	0.049 (0.033) *p* = 0.130
Supported you	5.185 (0.083)	5.181 (0.081)	**0.248** (0.042) *p* < 0.001	**0.281** (0.045) *p* < 0.001	0.084 (0.039) *p* = 0.033	**0.188** (0.039) *p* < 0.001
Criticized you	5.272 (0.085)	5.166 (0.084)	−**0.315** (0.062) *p* < 0.001	−**0.299** (0.049) *p* < 0.001	−0.086 (0.036) *p* = 0.021	−0.087 (0.058) *p* = 0.148

a *Random actor effects only. Bolded regression coefficients are significant at the p < 0.01 level*.

Actor effects showed associations between self-reported enacted communication and one's own RS. For both patients and spouses, providing support to one's partner was positively associated with one's own RS. Holding back from expressing one's feelings, in contrast, and criticizing one's partner, were negatively associated with one's own RS. With respect to perceived partner communication, for both patients and spouses, feeling supported was positively associated with one's own RS, and feeling criticized was negatively associated with one's own RS.

Partner effects were also found. For both patients and spouses, their own holding back was negatively associated with their partners' RS. In addition, spouses' support of the patient was positively associated with the patients' RS, while their criticism of the patient was negatively associated with the patient's RS. Lastly, when spouses *felt* supported, their patient partner reported higher RS.

### Lagged analyses

Table 5 displays coefficients, standard errors, and *p*-values from linear mixed models treating afternoon communication as the predictor and same-day evening RS as the criterion. As in the concurrent models, intercepts represent the adjusted sample means of RS for patients and spouses. Significant actor effects were found only for patients. For patients, supporting one's partner in the afternoon was associated with one's own higher RS that evening. For patients, there were also intrapersonal effects of feeling supported, and feeling criticized. If patients felt supported in the afternoon, their own RS was higher that evening. Conversely, if patients felt criticized in the afternoon, their own RS was lower that evening.

**Table 5:** Mixed model regression coefficients (standard errors) and *p*-values from lagged analyses: afternoon communication predicting evening relationship satisfaction.

	**Intercept**		**Actor effect**		**Partner effect**	
**Predictor**	**Patient**	**Spouse**	**Patient (a_11_)**	**Spouse (a_22_)**	**Patient (p_21_)**	**Spouse (p_12_)**
**To what extent did you…**						
Express your feelings	5.238 (0.098)	5.146 (0.102)	0.004 (0.031) *p* = 0.889	−0.004 (0.038) *p* = 0.909	−0.030 (0.038) *p* = 0.432	−0.013 (0.035) *p* = 0.706
Hold back from expressing your feelings	5.22 (0.090)	5.140 (0.091)	−0.040 (0.049) *p* = 0.427	−0.069 (0.054) *p* = 0.210	−0.021 (0.046) *p* = 0.649	−0.096 (0.054) *p* = 0.086
Support your partner	5.245 (0.093)	5.179 (0.088)	**0.146** (0.048) *p* = 0.003	0.115 (0.057) *p* = 0.048	0.098 (0.041) *p* = 0.026	0.076 (0.054) *p* = 0.168
Criticize your partner^a^	5.226 (0.098)	5.147 (0.090)	−0.101 (0.073) *p* = 0.179	−0.192 (0.073) *p* = 0.014	−**0.143** (0.052) *p* = 0.006	−0.104 (0.053) *p* = 0.050
**To what extent did you feel that your partner…**						
Expressed his/ her feelings	5.213 (0.098)	5.140 (0.101)	0.018 (0.043) *p* = 0.671	0.031 (0.045) *p* = 0.494	−0.007 (0.035) *p* = 0.845	0.005 (0.041) *p* = 0.911
Supported you^a^	5.213 (0.088)	5.184 (0.084)	**0.204** (0.049) *p* < 0.001	0.083 (0.047) *p* = 0.082	−0.021 (0.040) *p* = 0.603	0.077 (0.038) *p* = 0.044
Criticized you^a^	5.240 (0.090)	5.146 (0.090)	−**0.169** (0.050) *p* = 0.008	−0.138 (0.070) *p* = 0.055	−0.075 (0.048) *p* = 0.123	−**0.139** (0.044) *p* = 0.002

a *Random actor effects only. Bolded regression coefficients are significant at the p < 0.01 level*.

Partner effects were obtained for being critical (patients only) and feeling criticized (spouses only). If patients criticized their spouse in the afternoon, the spouse reported lower RS that same evening, and if spouses felt criticized by their patient in the afternoon, their patient reported lower RS that evening.

### Cross-lagged analyses

Table 6 displays coefficients and standard errors from linear mixed models treating evening RS as the predictor and next-day afternoon communication as the criterion. Here, each intercept represents the adjusted sample mean of the corresponding (afternoon) communication/perceived communication measure for either patients or spouses. As displayed in the upper section of Table 6, among spouses, evening RS was inversely associated with next-day afternoon holding back (actor effect). No other significant effects were found.

**Table 6:** Mixed model regression coefficients (standard errors) and *p*-values from cross-lagged analyses: evening relationship satisfaction predicting next-day afternoon communication.

	**Intercept**		**Actor effect**		**Partner effect**	
**Outcome**	**Patient**	**Spouse**	**Patient (a_11_)**	**Spouse (a_22_)**	**Patient (p_21_)**	**Spouse (p_12_)**
**To what extent did you…**						
Express your feelings^a^	3.344 (0.081)	3.196 (0.079)	0.032 (0.051) *p* = 0.535	0.026 (0.043) *p* = 0.550	0.069 (0.048) *p* = 0.151	0.067 (0.046) *p* = 0.147
Hold back from expressing your feelings^a^	1.460 (0.048)	1.462 (0.049)	−0.044 (0.040) *p* = 0.266	−**0.091** (0.032) *p* = 0.005	−0.030 (0.041) *p* = 0.470	−0.003 (0.037) *p* = 0.946
Support your partner^b^	3.978 (0.067)	3.941 (0.060)	0.083 (0.058) *p* = 0.160	−0.007 (0.040) *p* = 0.862	0.079 (0.037) *p* = 0.034	0.038 (0.039) *p* = 0.330
Criticize your partner^b^	1.238 (0.034)	1.256 (0.034)	−0.057 (0.037) *p* = 0.131	−0.008 (0.029) *p* = 0.789	0.004 (0.026) *p* = 0.890	−0.010 (0.028) *p* = 0.726
**To what extent did you feel that your partner…**						
Expressed his/ her feelings^c^	3.818 (0.074)	3.663 (0.068)	0.069 (0.045) *p* = 0.128	0.033 (0.041) *p* = 0.423	0.103 (0.042) *p* = 0.015	0.038 (0.044) *p* = 0.385
Supported you^b^	4.050 (0.057)	3.825 (0.064)	0.120 (0.046) *p* = 0.012	0.091 (0.044) *p* = 0.042	0.026 (0.038) *p* = 0.493	−0.012 (0.039) *p* = 0.762
Criticized you^b^	1.332 (0.037)	1.331 (0.044)	−0.049 (0.037) *p* = 0.194	−0.050 (0.032) *p* = 0.131	0.007 (0.031) *p* = 0.821	0.053 (0.032) *p* = 0.098

a Random partner effects only.

b Random actor effects only.

c *Actor and partner effects fixed. Bolded regression coefficients are significant at the p < 0.01 level*.

## Discussion

Results indicate that the nature and quality of communications between patients with cancer and their spouses were, as predicted, associated with concurrent ratings of RS and, to a lesser extent, later-day ratings of RS. The study used a heuristic framework based largely on the systemic transactional model, adapted to define communication broadly as not only stress-related, but also incorporating the construct of holding back from disclosure given its relevance to this population as demonstrated in prior studies (Porter et al., 2005; Edmond et al., 2013; Manne et al., 2014a). Using an ecological twice-daily assessment over 2 weeks, we were able to examine intra- and inter-personal effects of both enacted and perceived communication in concurrent (evening to same evening) and lagged (afternoon to same-day evening) scenarios. Importantly, this innovative smartphone-based assessment was feasible, with high rates of adherence including for those without smartphone experience who were loaned iPod Touch devices.

Across both the concurrent and lagged analyses, the most consistent effects were seen for ratings of one's own and one's partner's responsiveness. In concurrent analyses, for both patients and spouses, being supportive of their partner and feeling supported by their partner were associated with higher RS. Conversely, for both patients and spouses, being critical of their partner and feeling criticized by their partner were associated with lower RS. In lagged analyses, when patients reported supporting their spouse and feeling supported by their spouse, they later reported higher levels of RS. Conversely, when patients felt criticized by their spouse, they later reported lower levels of RS, and when they reported being critical, their spouse later reported lower levels of RS. These findings are commensurate with work on emotion-focused positive supportive dyadic coping and negative dyadic coping, respectively (Falconier et al., 2015), and extend this area to the realm of daily interactions of couples coping with cancer.

For both patients and spouses, and in both concurrent and lagged analyses, expressing one's own feelings was unassociated with RS. The one significant finding with regard to expression was that, in concurrent analyses, when patients perceived their partner as more expressive, their own RS was higher. This pattern of findings, combined with those above, suggests that disclosure *per se* may not be as crucial to RS as responses to the disclosure and how they are perceived. This is in line with results of a meta-analysis on dyadic coping and RS (Falconier et al., 2015). While disclosure may set the interaction in motion, the impact on the relationship appears to depend on the quality of the ensuing responses.

While expressiveness was largely unassociated with RS, holding back was inversely associated with RS as predicted. This finding was limited to the concurrent analyses and was the case for both actor and partner effects, and patients and spouses. In other words, when patients and spouses reported holding back from expressing their feelings, both they and their partners reported lower concurrent RS. These results are consistent with those obtained by Porter et al. (2005) using a traditional, global measure of holding back. Interestingly, the one significant finding from the cross-lagged analyses also involved holding back. When spouses reported lower levels of RS on 1 day, they were more likely to hold back from expressing feelings or concerns on the following day. Conversely, when spouses reported higher levels of RS on 1 day, they were less likely to hold back from expressing feelings or concerns on the following day. It makes sense that dissatisfaction with one's relationship on a given day might lead to reluctance to share thoughts and feelings on the following day. Clinically speaking, this suggests that attempts to bolster general satisfaction by fostering emotional closeness and intimacy and by creating opportunities for shared experiences could result in less holding back. To our knowledge, this is the first ecological study to examine temporal relationships between holding back and RS and the results suggest they may be reciprocal in nature. Taken together with previous research, these findings suggest that holding back may add explanatory power above the standard systemic transactional model, which does not address active withholding. The significant partner effects seen in the concurrent analyses and in previous research indicate that holding back is deleterious not just to the RS of the person who does it but also that of the partner. While the phrase “holding back” suggests a lack of emotional expression, the behavior may in fact be behaviorally manifest and observable to the partner. Indeed, laboratory research on expressive suppression, defined as “the conscious inhibition of emotional expressive behavior while emotionally aroused” (Gross and Levenson, 1993) indicates that this behavior disrupts communication and increases blood pressure in both those suppressing and their partners, and results in decreased rapport (Butler et al., 2003). This construct has been examined largely in the context of laboratory-based studies involving previously unacquainted undergraduate pairs where negative emotional experience is induced, and suppression is experimentally manipulated. This construct has been understudied both in the context of cancer and in naturalistic, day-to-day settings. Our findings indicate that this may be a fruitful area for further inquiry in couples coping with cancer and perhaps other stressful experiences as well.

Overall, the lagged analyses treating afternoon communication as the predictor and same-day evening RS as the criterion yielded fewer effects than concurrent analyses, and almost exclusively effects for patients. When patients provided support, and felt supported, they reported greater RS later that day. In contrast, when patients felt criticized, they reported lower RS later that day. These findings suggest that the carry-over effect of communication behavior, be that positive or negative, may be stronger for patients, perhaps due to the vulnerabilities associated with treatment and recovery. This finding bears replication, ideally in a fully mixed-gender patient sample. Research suggests that women are more interpersonally sensitive than are men (Acitelli, 1992; Lang-Takac and Osterweil, 1992; Lambert and Hopwood, 2016). Because our sample was comprised of more female than male patients (64.5% female), gender cannot be ruled out as a potential confound in attempts to explain any differences between patients and spouses.

While our predictions highlighted the primacy of communication in influencing RS, we also considered the possibility that there may be reciprocal relationships between these constructs such that they mutually influence each other over time. To test this, we examined RS in the evening as a predictor of next-day afternoon communication. As noted previously, these analyses yielded only one significant effect. In addition, regression coefficients were smaller in magnitude as compared to those for the lagged analyses. This suggests that, at least within the constraints of the paradigm used in this study, the influence of communication on ratings of RS may be stronger than the reverse. However, it should be noted that the cross-lagged analyses had a different time window (evening to next-day afternoon) than did the lagged (afternoon to same-day evening). Communication and RS may have been more closely connected in the lagged situation, in which the two variables were measured more proximally in time. In the cross-lagged situation, a full night and much of the following day separated ratings of RS and communication, and intervening events may have affected both variables in unknown ways.

The preponderance of findings for items designed to assess perceptions of partner behavior bear further consideration. Were such perceptions accurate? For example, if an individual reported being criticized by her partner, did the partner in fact report being critical? From our correlation matrices (Supplemental Tables 1, 2), we can infer that, for both patients and spouses, reports of feeling supported and feeling criticized were reflective of the way in which partners behaved. For example, patient report of feeling criticized in the afternoon was positively correlated with spouse report of having been critical at that same time (*r* = 0.39, *p* < 0.01), and spouse report of feeling criticized in the afternoon was positively correlated with patient report of having been critical at that same time (*r* = 0.29, *p* < 0.01). All eight relevant correlations (four for enacted and perceived support and criticism rated in the afternoon and four for enacted and perceived support and criticism rated in the evening) were positive in sign and statistically significant at the *p* < 0.01 level. This suggests that appraisals were in fact attuned to partner behavior (providing support for validity) and is commensurate with previous work demonstrating the value of partner-reports in couple research (Sanford, 2010).

In interpreting the present findings, it is important to note that levels of RS (rated on a 1–7 scale) were relatively high overall, certainly above the midpoint; see intercepts in Tables 4, 5. Similarly, levels of disclosure and support (rated on a 1–5 scale) were relatively high, and levels of holding back and criticism were relatively low (also rated on a 1–5 scale); see intercepts in Table 6. These findings are consistent with those from previous studies of cancer patients and partners (Porter et al., 2005; Manne et al., 2010). Couples who agree to be in a study on communication tend to be relatively well-adjusted. Indeed, the mean Dyadic Adjustment Scale scores for our sample fall within the non-distressed range (98–151) per standard cut-offs; see Table 2. Nonetheless, results of our regression analyses indicate important temporal patterns worthy of attention. Our findings suggest that couples' day-to-day interactions may influence their level of satisfaction with their relationship. Over time, these effects may accumulate, leading to overall declines in RS which could in turn affect mood and other important outcomes such as quality of life, pain, or physical functioning. Future work should consider more distal impacts of adaptive and maladaptive forms of communication. It would also be interesting to examine how these brief communication windows reflect patterns over a longer period of time and impact relationship stability. From the analyses reported, we cannot know whether the patterns seen reflect reactions to discrete events or represent longer term patterns of interaction among these couples.

Collectively, our findings are consistent with previous studies that point to the importance of conceptualizing cancer as a “we-disease” (Acitelli and Badr, 2005; Kayser et al., 2007) and bolster the need for joint approaches that treat both members of the couple as a source of support for the other, thereby co-facilitating adjustment (Bodenmann and Randall, 2013). Communication skills building is an essential part of this approach but, as noted by Badr (2017), simply prescribing more disclosure of emotions may not be sufficient. Rather, it may be necessary to address couples' motivations for avoiding cancer-related discussions, and to include training in skills for both expressing one's thoughts and feelings and listening to one's partner and responding in a supportive manner (Porter and Keefe, 2018). Our previous experience developing and testing such couple-based communication interventions suggest that they are feasible and acceptable (Porter et al., 2017; Langer et al., 2018), and that they lead to improvements in couples' relationship functioning (Porter et al., 2009, 2017). Interestingly, we have found that intervention effects are strongest among couples who report higher levels of holding back, providing further evidence of the potential importance of this variable (Porter et al., 2009). Studies using EMA methodology may provide a valuable adjunct to assessing intervention effects by examining communication and support processes as they unfold on a day-to-day basis, in the context of conversations around issues related to both cancer and other non-cancer topics.

Limitations of the present study must be considered. First, we only assessed RS once per day (in the evening), in part to minimize participant burden but also because we assumed that this variable would be relatively stable over the course of a day. Accordingly, we could not examine concurrent associations between communication and RS at the afternoon time point. Second, we did not assess conversational topic or valence of disclosures. We did, however, ask participants to rate the degree to which the topic was related to cancer and the importance of the conversation. Responses suggested that many conversations couples reported on may not have been directly cancer-related, but they were deemed relatively important. Thus, despite the rigors associated with treatment and recovery, couples frequently discuss important topics and concerns that may be unrelated to the cancer. It is unclear whether, in the context of cancer, there may be different communication patterns when discussing cancer vs. non-cancer topics, and whether these are differentially important for couples' RS. It is likely that couples infrequently engage in conversations specifically focused on the cancer in the absence of precipitating events, such as a doctor's visit or receipt of test results. Thus, studies using EMA methods may have difficulty capturing such conversations. Future studies could be designed to assess couples around the time of such events or could assess couples over multiple time periods (e.g., 1 week/month over several months) to increase the likelihood of capturing important cancer-related conversations. Future studies might also incorporate more specific questions about the topic of conversations and valence (positivity or negativity) of disclosures. A third limitation of this study is that our analyses were limited to same and next-day effects. Examining longer time lags may yield important information regarding the degree to which communication behaviors affect RS over time. Lastly, the fact that the couples in this study were relatively well-adjusted may limit generalizability.

Despite these limitations, the present study has several notable strengths. First, the sample size was fairly large for this population and, unlike much of the literature on couple communication in cancer, includes a subset of patients coping with advanced disease (64%). Second, our sample includes both male and female patients and was drawn from two different geographical regions across the United States. Third, our response rate was quite high (90%), as was our completion rate (89%), thus demonstrating that smartphone-enabled EMA is feasible, even for an arguably stressed and vulnerable population. Fourth, the inclusion of reports not just of one's own behavior but of perceptions of one's partner's behavior add significantly to our understanding of the role of appraisal in the context of dyadic coping.

In conclusion, findings from this EMA study underscore the importance of responsive communication in explaining day-to-day relationship adjustment among patients with cancer and their caregiving partners. These findings confirm predictions of the systemic transactional model with regard to emotion-focused positive supportive dyadic coping and negative dyadic coping (Bodenmann, 2005). They also extend the model to include the construct of holding back as potentially important in understanding RS for couples coping with cancer.

## Author contributions

SL, LP, JR, FK, TS, KS, JWB, JBB, NB, JG, VS, KW, and SZ secured funding for the study. SL, LP, JR, FK, TS, KS, JWB, JBB, and NB contributed to study conception and design. NG and BP recruited and consented participants, trained participants in the smartphone application, and tracked data collection. JG, VS, KW, and SZ supported and facilitated patient recruitment. MT performed statistical analyses. SL, MT, LP, JR, TS, and NG wrote the first draft of the manuscript. All authors reviewed and approved the submitted version.

### Conflict of interest statement

The authors declare that the research was conducted in the absence of any commercial or financial relationships that could be construed as a potential conflict of interest.
