# Supplementary material for: Links Between Communication and Relationship Satisfaction Among Patients With Cancer and Their Spouses: Results of a Fourteen-Day Smartphone-Based Ecological Momentary Assessment Study
Source: Front Psychol. 2018 Oct 10;9:1843. doi: 10.3389/fpsyg.2018.01843 (PMC6191515; doi:10.3389/fpsyg.2018.01843)
Supplement: Supplementary file 1 [file Table_1.docx]

| Table 1. Correlations among relationship satisfaction (RS) ratings and afternoon reports of communication | | | | | | | | | | | | | | | | | |
| --- | --- | --- | --- | --- | --- | --- | --- | --- | --- | --- | --- | --- | --- | --- | --- | --- | --- |
|  | Variable | 1 | 2 | 3 | 4 | 5 | 6 | 7 | 8 | 9 | 10 | 11 | 12 | 13 | 14 | 15 | 16 |
| 1. | PT RS | - |  |  |  |  |  |  |  |  |  |  |  |  |  |  |  |
| 2. | SP RS | .39^**^ | - |  |  |  |  |  |  |  |  |  |  |  |  |  |  |
| 3. | PT expressed | .01 | -.02 | - |  |  |  |  |  |  |  |  |  |  |  |  |  |
| 4. | PT held back | -.02 | -.03 | -.05 | - |  |  |  |  |  |  |  |  |  |  |  |  |
| 5. | PT supported | .10^**^ | .05 | .20^**^ | -.16^**^ | - |  |  |  |  |  |  |  |  |  |  |  |
| 6. | PT criticized | -.04 | -.09^*^ | .11^**^ | .27^**^ | -.22^**^ | - |  |  |  |  |  |  |  |  |  |  |
| 7. | PT-rated SP express | .04 | -.01 | .32^**^ | -.05 | .30^**^ | .00 | - |  |  |  |  |  |  |  |  |  |
| 8. | PT felt supported | .10^**^ | .02 | .22^**^ | -.28^**^ | .46^**^ | -.27^**^ | .29^**^ | - |  |  |  |  |  |  |  |  |
| 9. | PT felt criticized | -.09^**^ | -.10^**^ | .03 | .22^**^ | -.21^**^ | .48^**^ | .08^*^ | -.40^**^ | - |  |  |  |  |  |  |  |
| 10. | SP expressed | -.02 | .01 | .14^**^ | .08^*^ | .07^*^ | .09^**^ | .08^*^ | .03 | .09^**^ | - |  |  |  |  |  |  |
| 11. | SP held back | -.06 | -.04 | .01 | .10^**^ | -.09^**^ | .10^**^ | -.05 | -.13^**^ | .11^**^ | -.07^*^ | - |  |  |  |  |  |
| 12. | SP supported | .07^*^ | .09^*^ | .02 | .01 | .09^**^ | -.08^*^ | .03 | .12^**^ | -.13^**^ | .19^**^ | -.11^**^ | - |  |  |  |  |
| 13. | SP criticized | -.06 | -.09^**^ | -.02 | .13^**^ | -.15^**^ | .18^**^ | .01 | -.28^**^ | .39^**^ | .07^*^ | .23^**^ | -.25^**^ | - |  |  |  |
| 14. | SP-rated PT express | .00 | .03 | .10^**^ | .02 | .06 | -.01 | .11^**^ | .10^**^ | -.05 | .31^**^ | -.05 | .24^**^ | -.02 | - |  |  |
| 15. | SP felt supported | .03 | .08^*^ | .15^**^ | -.11^**^ | .24^**^ | -.16^**^ | .08^*^ | .26^**^ | -.21^**^ | .18^**^ | -.23^**^ | .48^**^ | -.28^**^ | .32^**^ | - |  |
| 16. | SP felt criticized | -.04 | -.08^*^ | .03 | .15^**^ | -.18^**^ | .29^**^ | .01 | -.25^**^ | .28^**^ | .06^*^ | .32^**^ | -.26^**^ | .53^**^ | .01 | -.35^**^ | - |
| *Note*. **p* < .05, ***p* < .01. PT = patient and SP = spouse. PT-rated SP express and SP-rated PT express refer to patient and spouse perceptions, respectively, of the extent to which their partner expressed his/her feelings. | | | | | | | | | | | | | | | | | |
