# Supplementary material for: Links Between Communication and Relationship Satisfaction Among Patients With Cancer and Their Spouses: Results of a Fourteen-Day Smartphone-Based Ecological Momentary Assessment Study
Source: Front Psychol. 2018 Oct 10;9:1843. doi: 10.3389/fpsyg.2018.01843 (PMC6191515; doi:10.3389/fpsyg.2018.01843)
Supplement: Supplementary file 2 [file Table_2.docx]

| Table 2. Correlations among relationship satisfaction (RS) ratings and evening reports of communication | | | | | | | | | | | | | | | | | |
| --- | --- | --- | --- | --- | --- | --- | --- | --- | --- | --- | --- | --- | --- | --- | --- | --- | --- |
|  | Variable | 1 | 2 | 3 | 4 | 5 | 6 | 7 | 8 | 9 | 10 | 11 | 12 | 13 | 14 | 15 | 16 |
| 1. | PT RS | - |  |  |  |  |  |  |  |  |  |  |  |  |  |  |  |
| 2. | SP RS | .39^**^ | - |  |  |  |  |  |  |  |  |  |  |  |  |  |  |
| 3. | PT expressed | .04 | .03 | - |  |  |  |  |  |  |  |  |  |  |  |  |  |
| 4. | PT held back | -.11^**^ | -.09^**^ | .01 | - |  |  |  |  |  |  |  |  |  |  |  |  |
| 5. | PT supported | .16^**^ | .09^**^ | .18^**^ | -.10^**^ | - |  |  |  |  |  |  |  |  |  |  |  |
| 6. | PT criticized | -.17^**^ | -.11^**^ | .12^**^ | .14^**^ | -.20^**^ | - |  |  |  |  |  |  |  |  |  |  |
| 7. | PT-rated SP express | .07^*^ | .01 | .32^**^ | -.01 | .35^**^ | -.04 | - |  |  |  |  |  |  |  |  |  |
| 8. | PT felt supported | .19^**^ | .14^**^ | .17^**^ | -.21^**^ | .48^**^ | -.30^**^ | .27^**^ | - |  |  |  |  |  |  |  |  |
| 9. | PT felt criticized | -.19^**^ | -.09^**^ | .03 | .20^**^ | -.28^**^ | .47^**^ | -.02 | -.45^**^ | - |  |  |  |  |  |  |  |
| 10. | SP expressed | .00 | .03 | .14^**^ | -.01 | .04 | .02 | .14^**^ | .03 | .04 | - |  |  |  |  |  |  |
| 11. | SP held back | -.05 | -.14^**^ | .03 | .03 | -.08^*^ | .07^*^ | .00 | -.07^*^ | .05 | -.01 | - |  |  |  |  |  |
| 12. | SP supported | .06^*^ | .14^**^ | .07^*^ | -.05 | .11^**^ | -.11^**^ | .06^*^ | .22^**^ | -.18^**^ | .21^**^ | -.11^**^ | - |  |  |  |  |
| 13. | SP criticized | -.04 | -.11^**^ | .02 | .03 | -.09^**^ | .11^**^ | .03 | -.16^**^ | .26^**^ | .18^**^ | .13^**^ | -.23^**^ | - |  |  |  |
| 14. | SP-rated PT express | .02 | .06^*^ | .11^**^ | -.01 | .02 | .01 | .11^**^ | .05 | .03 | .27^**^ | -.02 | .26^**^ | -.02 | - |  |  |
| 15. | SP felt supported | .07^*^ | .17^**^ | .04 | -.02 | .16^**^ | -.14^**^ | .08^*^ | .20^**^ | -.17^**^ | .14^**^ | -.23^**^ | .46^**^ | -.28^**^ | .25^**^ | - |  |
| 16. | SP felt criticized | -.05 | -.15^**^ | .04 | .09^**^ | -.17^**^ | .29^**^ | .02 | -.20^**^ | .24^**^ | .10^**^ | .19^**^ | -.22^**^ | .38^**^ | .00 | -.36^**^ | - |
| *Note*. **p* < .05, ***p* < .01. PT = patient and SP = spouse. PT-rated SP express and SP-rated PT express refer to patient and spouse perceptions, respectively, of the extent to which their partner expressed his/her feelings. | | | | | | | | | | | | | | | | | |
